# Restoration of Developmental Competence in Low-Quality Porcine Cumulus–Oocyte Complexes through the Supplementation of Sonic Hedgehog Protein during In Vitro Maturation

**DOI:** 10.3390/ani13061001

**Published:** 2023-03-09

**Authors:** Pil-Soo Jeong, Hyo-Gu Kang, Bong-Seok Song, Sun-Uk Kim, Bo-Woong Sim, Sanghoon Lee

**Affiliations:** 1 Futuristic Animal Resource & Research Center, Korea Research Institute of Bioscience and Biotechnology, Cheongju 28116, Republic of Korea; 2 Department of Functional Genomics, KRIBB School of Bioscience, Korea University of Science and Technology, Daejeon 34113, Republic of Korea; 3 Laboratory of Theriogenology, College of Veterinary Medicine, Chungnam National University, Daejeon 34134, Republic of Korea

**Keywords:** sonic hedgehog signaling, oocyte quality, brilliant cresyl blue, cumulus cell expansion, oocyte maturation, pig

## Abstract

**Simple Summary:**

Oocyte quality is acquired during ovarian folliculogenesis including oocyte growth and maturation, and plays a key role in subsequent embryo development. However, oocytes matured in vitro do not fully support the ability to develop into the blastocyst stage due to inadequate in vitro maturation (IVM) systems which do not sufficiently mimic the in vivo microenvironment. The sonic hedgehog (SHH) signaling pathway is important for ovarian folliculogenesis and oocyte maturation. Furthermore, our previous study demonstrated that low-quality porcine cumulus–oocyte complexes (COCs) exhibit low developmental competence with a weak SHH signaling pathway. Therefore, the aim of the present study was to clarify the restorative effects of SHH protein supplementation during IVM on the developmental competence of low-quality porcine COCs, as assessed by brilliant cresyl blue staining. The results showed that the low developmental competence of low-quality porcine COCs can be improved by supplementation with the SHH protein. This indicates that an active SHH signaling pathway is required for the acquisition of developmental competence in porcine COCs.

**Abstract:**

The sonic hedgehog (SHH) pathway is an important signaling pathway for mammalian ovarian folliculogenesis and oocyte maturation. A previous study demonstrated that low-quality porcine cumulus–oocyte complexes (COCs) have low developmental competence, with lower SHH signaling protein expression before and after in vitro maturation (IVM) than high-quality COCs. However, there is no reported evidence on the restorative effects of SHH protein supplementation during the IVM of low-quality porcine COCs. Therefore, this study investigated the effects of SHH protein supplementation on the IVM of low-quality porcine COCs, as assessed by brilliant cresyl blue (BCB) staining. To examine this, we designed four groups: (i) BCB− (low-quality), (ii) BCB− + SHH, (iii) BCB+ (high-quality), and (iv) BCB+ + SHH. While the supplementation of SHH protein with high-quality COCs had no effect, supplementation with low-quality COCs significantly improved cumulus cell expansion, metaphase II rate, and subsequent embryo development following parthenogenetic activation. Our results provide the first evidence that the low developmental competence of low-quality porcine COCs can be improved by supplementation with the SHH protein. These results indicate that an active SHH signaling pathway is required for the acquisition of developmental competence in porcine COCs.

## 1. Introduction

The in vitro maturation (IVM) of oocytes is a useful technique for successful in vitro fertilization and embryo production. However, the developmental potential of IVM oocytes is lower than that of in vivo-matured oocytes. Morphological criteria, such as the number of surrounding cumulus cell layers and ooplasm homogeneity, are usually used for IVM [1]. However, these criteria are sometimes inconsistent and unreliable [2].

Brilliant cresyl blue (BCB) staining has recently been used to select developmentally competent oocytes before IVM. This is based on the assessment of different glucose-6-phosphate dehydrogenase (G6PDH) reactions, which convert the BCB dye from blue to colorless [3]. G6PDH activity is high in the cytoplasm of developing oocytes and reduces in fully grown oocytes [4]. This approach can be used to distinguish the more developmentally competent BCB-stained (BCB+) oocytes (low G6PDH activity) from the less competent BCB non-stained (BCB−) oocytes (high G6PDH activity). BCB staining is a reliable method for selecting cumulus–oocyte complexes (COCs) without adversely affecting oocyte quality in mammals, such as mice, cattle, goats, and pigs [5]. Multiple molecular and physical differences exist between BCB+ and BCB− oocytes, such as diameter, lipid content, mitochondrial function, and gene expression [6]. Notably, BCB+ oocytes exhibit significantly higher in vitro embryo development than BCB− oocytes, indicating that the quality of BCB− oocytes is lower than that of BCB+ oocytes [7,8].

The evolutionarily conserved sonic hedgehog (SHH) signaling pathway plays a crucial role in the proliferation, differentiation, and survival of various cell types via paracrine signaling [9]. Transduction of the SHH signaling pathway occurs through binding of the secreted SHH ligand to the transmembrane receptor Patched (PTCH). This relieves the inhibition of the Smoothened (SMO) protein and subsequently activates the nuclear translocation of the transcription factor glioma-associated oncogene homolog 1 (GLI1) to regulate target gene expression [10,11]. The SHH signaling pathway is involved in the growth and development of follicles in the mammalian ovary. Additionally, recombinant SHH protein treatment increases the growth and proliferation of granulosa cells in mice, goats, and cattle [12,13,14]. Moreover, SHH signaling pathway-related genes and proteins are expressed in the cumulus cell layer of oocytes, and supplementation with recombinant SHH protein during IVM significantly improves oocyte maturation and subsequent embryo development in pigs [15].

Our previous study reported that high-quality porcine COCs exhibit a high proportion of fully expanded cumulus cells, nuclear maturation of oocytes, and subsequent embryo development with a high expression of SHH signaling pathway-related proteins. Alternatively, low-quality COCs have a relatively low expression of SHH signaling pathway-related proteins [8]. However, no study investigated the restorative effects of SHH protein supplementation during the IVM of low-quality porcine COCs. Therefore, this study investigated such effects on the developmental competence of low and high-quality porcine COCs, as assessed by BCB staining. We sought to elucidate and confirm the relationship between oocyte quality and the SHH signaling pathway.

## 2. Materials and Methods

### 2.1. Chemicals

All chemicals and reagents were purchased from Sigma-Aldrich Chemical Co. (St. Louis, MO, USA) unless otherwise indicated.

### 2.2. Isolation and IVM of Porcine COCs

Porcine ovaries were collected from a local slaughterhouse and transported to the laboratory in a sterile 0.9% NaCl solution. The COCs were recovered from ovarian follicles sized 3–6 mm by aspiration with an 18-gauge needle and a 10-mL syringe. Only COCs with a minimum of three cumulus cell layers were used for IVM. Before IVM, the COCs were classified as BCB− or BCB+, according to their coloration through BCB staining. Then, BCB− or BCB+ COCs were cultured with or without 0.5 µg/mL recombinant mouse SHH protein (SHH N-Terminus; 461-SH-025; R&D Systems, Minneapolis, MN, USA) during the entire period of IVM. The concentration of SHH protein was set according to a previous study [15]. The IVM medium was composed of tissue culture medium-199 supplemented with 10 ng/mL epidermal growth factor, 10 ng/mL β-mercaptoethanol, 0.57 mM cysteine, 10% porcine follicular fluid, 10 IU/mL human chorionic gonadotropin (Prospec, East Brunswick, NJ, USA), and 10 IU/mL pregnant mare’s serum gonadotropin (Prospec). The COCs were cultured in IVM medium under 5% CO_2_ at 38.5 °C for 22 h. They were then washed and cultured in hormone-free IVM medium for an additional 22 h.

### 2.3. Evaluation of Cumulus Cell Expansion

Cumulus cell expansion after 44 h of IVM was evaluated by morphological examination using a stereomicroscope (Nikon Corp., Tokyo, Japan), according to previous studies [8,16]: Degree 0, no visible expansion; degree 1, minor expansion with a spherical appearance of the cumulus cells; degree 2, expansion of outermost layers of the cumulus cells; degree 3, expansion of all cumulus cell layers except the corona radiata; degree 4, expansion of all cumulus cell layers including the corona radiata.

### 2.4. Evaluation of Nuclear Maturation of Oocytes

Cumulus cells after 44 h of IVM were removed by gentle pipetting in PB1 medium [0.4% bovine serum albumin (BSA) in Dulbecco’s phosphate-buffered saline (DPBS; Gibco, Grand Island, NY, USA)] containing 0.1% hyaluronidase. According to previous studies [17,18], denuded oocytes were considered as metaphase II (MII), immature, or degenerate using a stereomicroscope (Nikon Corp.)

### 2.5. Parthenogenetic Activation (PA) of Oocytes

The MII oocytes were gradually equilibrated in 280 mM mannitol solution containing 0.1 mM CaCl_2_·2H_2_O, 0.1 mM MgSO_4_·7H_2_O, 0.5 mM HEPES, and 0.01% polyvinyl alcohol (PVA). Oocytes were activated by a single direct current pulse (1.1 kV/cm for 50 μs) using an Electro Cell Fusion Generator (LF 101; Nepa Gene, Chiba, Japan), according to a previous study [19]. The electrically-activated oocytes were transferred into post-activation medium [porcine zygote medium-3 (PZM-3)] supplemented with 5 μg/mL cytochalasin B and 2 mM 6-dimethylaminopurine under 5% CO_2_ at 38.5 °C for 4 h. Then, the electrically-activated oocytes were washed and cultured in PZM-3 under 5% CO_2_ at 38.5 °C for 6 days.

### 2.6. CDX2 Staining by Immunocytochemistry

For fixation, blastocysts were incubated in 4% paraformaldehyde longer than 24 h at 4 °C. For permeabilization, blastocysts were incubated in 1% Triton X-100 in DPBS at room temperature for 1 h. After blastocysts were washed three times in DPBS supplemented with 0.1% PVA (DPBS-PVA), they were blocked in 1% BSA in DPBS-PVA (DPBS-PVA-BSA) overnight at 4 °C and further incubated in 10% normal goat serum for 1 h. Then, blastocysts were incubated with a mouse monoclonal CDX2 antibody (AM392; Biogenex, San Ramon, CA, USA) overnight at 4 °C. After three times washing in DPBS-PVA-BSA, blastocysts were incubated with conjugated secondary antibodies, Alexa Fluor 488-labeled anti-mouse IgG (1:200) at room temperature for 1 h. After three times of washing in DPBS-PVA-BSA, blastocysts were mounted on clean glass slides with a Vectashield mounting medium (Vector Laboratories, Burlingame, CA, USA) containing 2 µg/mL DAPI. DAPI-labeled or CDX2-positive nuclei were examined under a fluorescence microscope (Leica DMi8; Leica Microsystems, Wetzlar, Germany).

### 2.7. TUNEL Assay

The apoptosis levels in blastocysts were examined by terminal deoxynucleotidyl transferase mediated dUTP digoxygenin nick end labeling (TUNEL) assay, according to previous studies [20,21]. For fixation, blastocysts were incubated in 4% paraformaldehyde at 4 °C longer than 24 h. For permeabilization, blastocysts were incubated in 1% Triton X-100 in DPBS at room temperature for 1 h. After three times of washing in DPBS-PVA, nonspecific binding sites were blocked by incubating in DPBS containing 2% BSA for 1 h. Blastocysts were incubated in TUNEL reaction medium (11684795910; Roche, Basel, Switzerland) for 1 h at 38.5 °C. Next, blastocysts were washed three times with DPBS-PVA for 10 min and then mounted on clean glass slides with a Vectashield mounting medium containing 2 µg/mL DAPI (Vector Laboratories). The number of total cells (blue) or apoptotic cells (green) per blastocyst were examined under a fluorescence microscope (Leica DMi8; Leica Microsystems).

### 2.8. Statistical Analysis

Statistical analyses were performed using SigmaStat statistical program (SPSS, Inc., Chicago, IL, USA). All data were subjected to normality and homoscedasticity test. Then, the Kruskal–Wallis test (for data with a non-normal distribution) and one-way ANOVA (for data with a normal distribution) were used to identify significant differences. One-way ANOVA was followed by Duncan’s multiple range test (for equal variance) or Dunnett’s T3 test (for unequal variance). Each experiment consisted of at least three replicates and data were expressed as means ± standard error of the mean. *p*-values < 0.05 were considered to denote statistical significance.

## 3. Results

### 3.1. Effect of SHH Protein Supplementation during Porcine IVM on Cumulus Cell Expansion of BCB− and BCB+ COCs

**Figure 1:**
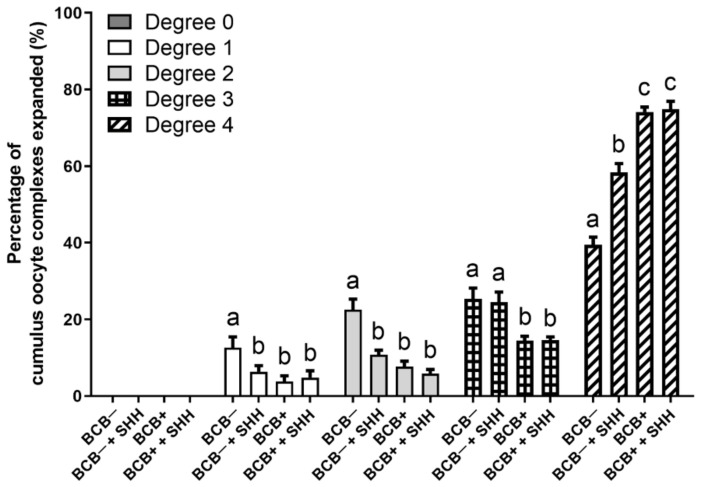
Effect of sonic hedgehog (SHH) protein supplementation during in vitro maturation of brilliant cresyl blue (BCB)− and BCB+ cumulus–oocyte complexes (COCs) on cumulus cell expansion. The experiment was independently replicated four times with at least 100 COCs per group. Groups indicated by different letters (a–c) represent significant differences (*p* < 0.05). BCB−, BCB non-stained COCs; BCB− + SHH, BCB− COCs supplemented with 0.5 µg/mL SHH protein; BCB+, BCB stained COCs; BCB+ + SHH, BCB+ COCs supplemented with 0.5 µg/mL SHH protein.

Prior to IVM, the porcine COCs were classified as BCB− or BCB+, based on their coloration using BCB staining. BCB− and BCB+ COCs were cultured with or without 0.5 µg/mL recombinant mouse SHH protein during the IVM period. After 44 h, the cumulus cell expansion in each group was investigated (Figure 1). A degree of 0 was not observed in any group. The proportions of degrees 1 and 2 were significantly decreased with SHH supplementation of BCB− COCs compared to those in the BCB− group, which was comparable to those of the BCB+ and BCB+ + SHH groups. For degree 3, SHH supplementation of BCB− COCs showed no significant difference in proportion compared to the BCB− group, which was significantly higher than in the BCB+ and BCB+ + SHH groups. For degree 4, SHH supplementation of BCB− COCs resulted in a significant increase in its proportion compared to the BCB− group. However, the proportion of degree 4 in the BCB− + SHH group was still significantly lower than in the BCB+ and BCB+ + SHH groups.

### 3.2. Effect of SHH Protein Supplementation during Porcine IVM on Oocyte Nuclear Maturation of BCB− and BCB+ COCs

**Figure 2:**
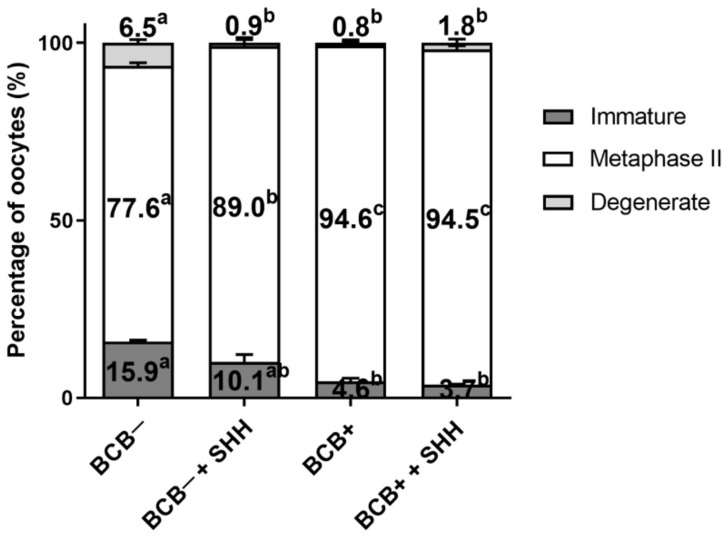
Effect of sonic hedgehog (SHH) protein supplementation during in vitro maturation of brilliant cresyl blue (BCB)− and BCB+ cumulus–oocyte complexes (COCs) on oocyte nuclear maturation. The experiment was independently replicated four times with at least 100 oocytes per group. Groups indicated by different letters (a–c) represent significant differences (*p* < 0.05). BCB−, BCB non-stained COCs; BCB− + SHH, BCB− COCs supplemented with 0.5 µg/mL SHH protein; BCB+, BCB stained COCs; BCB+ + SHH, BCB+ COCs supplemented with 0.5 µg/mL SHH protein.

The effect of SHH supplementation on oocyte nuclear maturation in BCB− and BCB+ COCs was investigated (Figure 2). SHH supplementation of BCB− COCs showed no significant difference in immature rate compared to the BCB− group. However, the degeneration rate was significantly decreased upon SHH supplementation of BCB− COCs compared to the BCB− group, which was comparable to those of the BCB+ and BCB+ + SHH groups. For the MII rate, the proportion was significantly increased upon SHH supplementation of BCB− COCs compared to the BCB− group. However, the MII rate in the BCB− + SHH group was still significantly lower than the BCB+ and BCB+ + SHH groups.

### 3.3. Effect of SHH Protein Supplementation during Porcine IVM on the Subsequent Development of PA Embryos Derived from BCB− and BCB+ COCs

**Figure 3:**
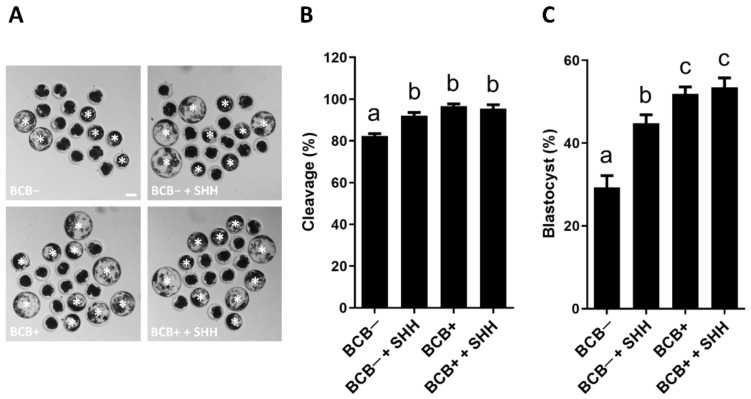
Effect of sonic hedgehog (SHH) protein supplementation during in vitro maturation of brilliant cresyl blue (BCB)− and BCB+ cumulus–oocyte complexes (COCs) on subsequent embryo development after parthenogenetic activation. (**A**) Representative photomicrographs of blastocysts (white asterisks). (**B**) The cleavage rates in the indicated groups. (**C**) The blastocyst formation rates in the indicated groups. The experiment was independently replicated four times with at least 70 embryos per group. Groups indicated by different letters (a–c) represent significant differences (*p* < 0.05). BCB−, BCB non-stained COCs; BCB− + SHH, BCB− COCs supplemented with 0.5 µg/mL SHH protein; BCB+, BCB stained COCs; BCB+ + SHH, BCB+ COCs supplemented with 0.5 µg/mL SHH protein. Bar = 100 μm.

The effect of SHH supplementation during IVM of BCB− and BCB+ COCs on the PA embryo development was investigated (Figure 3). SHH supplementation of BCB− COCs significantly increased the cleavage rate compared to the BCB− group, which was comparable to the BCB+ and BCB+ + SHH groups. Similarly, SHH supplementation of BCB− COCs significantly increased the blastocyst formation rate compared to the BCB− group. However, it was significantly lower than in the BCB+ and BCB+ + SHH groups.

### 3.4. Effect of SHH Protein Supplementation during Porcine IVM on Quality and Apoptosis Levels in PA Blastocysts Derived from BCB− and BCB+ COCs

**Figure 4:**
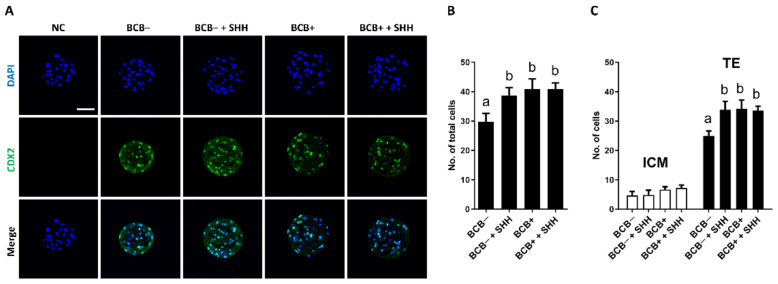
Effect of sonic hedgehog (SHH) protein supplementation during in vitro maturation of brilliant cresyl blue (BCB)− and BCB+ cumulus–oocyte complexes (COCs) on cell numbers of parthenogenetic blastocysts. (**A**) Representative photomicrographs of CDX2 immunocytochemical staining of parthenogenetic blastocysts developed from the indicated groups. Quantification of (**B**) the total, (**C**) inner cell mass (ICM), and trophectoderm (TE) cell numbers in the indicated groups. The experiment was independently replicated three times with at least 7 blastocysts per group. Groups indicated by different letters (a and b) represent significant differences (*p* < 0.05). NC, negative control; BCB−, BCB non-stained COCs; BCB− + SHH, BCB− COCs supplemented with 0.5 µg/mL SHH protein; BCB+, BCB stained COCs; BCB+ + SHH, BCB+ COCs supplemented with 0.5 µg/mL SHH protein. Bar = 100 μm.

**Figure 5:**
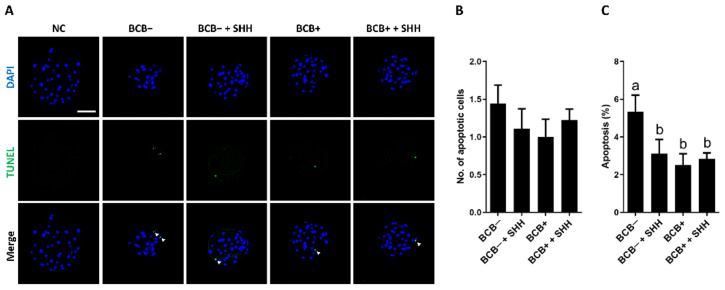
Effect of sonic hedgehog (SHH) protein supplementation during in vitro maturation of brilliant cresyl blue (BCB)− and BCB+ cumulus–oocyte complexes (COCs) on apoptosis levels in parthenogenetic blastocysts. (**A**) Representative photomicrographs of TUNEL staining of parthenogenetic blastocysts in the indicated groups. Quantification of (**B**) numbers and (**C**) proportion of apoptotic cells in the indicated groups. The experiment was independently replicated three times with at least 9 blastocysts per group. Groups indicated by different letters (a and b) represent significant differences (*p* < 0.05). NC, negative control; BCB−, BCB non-stained COCs; BCB− + SHH, BCB− COCs supplemented with 0.5 µg/mL SHH protein; BCB+, BCB stained COCs; BCB+ + SHH, BCB+ COCs supplemented with 0.5 µg/mL SHH protein. Bar = 100 μm.

The effect of SHH supplementation on the quality and apoptosis levels in PA blastocysts derived from BCB− and BCB+ COCs was investigated. In terms of blastocyst quality (Figure 4), SHH supplementation of BCB− COCs significantly increased the total and trophectoderm (TE) cell numbers in blastocysts compared to the BCB− group. The results of that group were comparable to those of the BCB+ and BCB+ + SHH groups. However, no significant difference was observed in the number of inner cell mass (ICM) cells among the groups. In terms of apoptosis levels in blastocysts (Figure 5), SHH supplementation of BCB− COCs significantly decreased the percentage of apoptotic cells in blastocysts compared to the BCB− group, which was similar to that of the BCB+ and BCB+ + SHH groups. However, no difference was found in the number of apoptotic cells in the blastocysts among the groups.

## 4. Discussion

In the present study, the restorative effects of SHH protein supplementation during the IVM of low-quality porcine COCs were investigated. SHH supplementation of BCB− COCs (low-quality) improved cumulus cell expansion, oocyte nuclear maturation, and subsequent PA embryo development compared to the non-supplemented BCB− group, although some of them were still not comparable to the BCB+ group (high-quality). Our previous study demonstrated that high-quality porcine COCs show a high developmental competence with an active SHH signaling pathway, while low-quality porcine COCs exhibit a low developmental competence with a weak SHH signaling pathway. The results of the present study confirmed that the low developmental competence of low-quality porcine COCs can be improved by SHH activation through supplementation with SHH protein. Therefore, from these results, it can be speculated that the SHH signaling pathway is strongly related to oocyte quality and an active SHH signaling pathway is required for the acquisition of developmental competence in porcine COCs.

Considering the in vivo physiology of oocyte maturation, differences in developmental competence based on the quality of COCs may be closely related to cumulus cell expansion. During ovarian follicular development, cumulus cells surrounding the oocyte play an important role in oocyte growth and maturation by providing many factors, including nutrients, messenger molecules, and hormones [22,23]. As the optimum expansion of cumulus cells is required for proper oocyte maturation and acquisition of developmental competence, cumulus cell expansion is considered a gross indicator of oocyte maturation [24]. The SHH signaling pathway is involved in ovarian follicular development and cumulus cell expansion in mammals [12,25]. In addition, genes and proteins of SHH, PTCH, SMO, and GLI exist in the porcine cumulus cell layer, indicating that the SHH signaling pathway is related to cumulus cell expansion in porcine COCs [15,26]. In our previous study, we confirmed that BCB+ COCs exhibit a significantly higher proportion of complete cumulus cell expansion with highly expressed SHH signaling-related proteins compared to BCB− COCs. This indicated that high-quality COCs are more likely to expand their surrounding cumulus cells with an active SHH signaling pathway than low-quality COCs [8]. Furthermore, the present study showed that SHH supplementation of BCB− COCs significantly enhanced cumulus cell expansion compared to the BCB− group. These results suggest that the SHH signaling pathway is closely related to cumulus cell expansion in porcine COCs, and activation of the SHH signaling pathway may play a beneficial role in improving the cumulus cell expansion of low-quality porcine COCs.

Recently, it has been reported that the SHH signaling pathway is associated with oocyte nuclear maturation. SHH protein supplementation during porcine and caprine IVM significantly increases the MII rate by regulating cyclin B1 content and mitogen-activated protein kinase phosphorylation, which play critical roles in the meiotic process [13,15]. In addition, our previous study found that BCB+ porcine COCs exhibit a significantly higher MII rate with highly expressed SHH signaling-related proteins than BCB− COCs [8]. In this study, we confirmed that SHH protein supplementation of BCB− COCs significantly increased the MII rate compared to the BCB− group. These results indicate that the SHH signaling pathway is essential for oocyte nuclear maturation, and the low MII rate in low-quality porcine COCs can be improved by activating the SHH signaling pathway.

Oocyte quality, which is gradually acquired during ovarian folliculogenesis, including oocyte growth and maturation, plays a key role in subsequent embryo development, pregnancy maintenance, and fetal development [27,28]. However, only a small percentage of IVM oocytes can develop to the blastocyst stage and subsequent pregnancy, indicating that IVM systems do not sufficiently support the developmental competence of oocytes [29]. Thus, improving oocyte quality during IVM is important for successful embryonic development. This can be achieved by mimicking the in vivo environment of the ovary to promote oocyte maturation in vitro, such as by activating the SHH signaling pathway during IVM. Previous studies have demonstrated that SHH protein supplementation during IVM increases oocyte quality and subsequent embryo development in pigs and goats [13,15]. Moreover, previous studies have demonstrated that melatonin and resveratrol improve oocyte quality and subsequent embryo development by activating the SHH signaling pathway [26,30]. Consistent with previous studies, we confirmed that SHH protein supplementation significantly increased the developmental competence of BCB− COCs compared to the non-supplemented BCB− group. Based on these results, it was demonstrated that activation of the SHH signaling pathway during IVM could help the restoration of developmental competence in low-quality porcine COCs.

## 5. Conclusions

For the first time, we confirmed that the low developmental competence of low-quality porcine COCs can be improved by supplementation with the SHH protein. This indicates that the active SHH signaling pathway is crucial for the acquisition of developmental competence in porcine COCs. Our findings provide insights into the role of the SHH signaling pathway in porcine oocyte maturation and will be useful for the development of new porcine IVM systems.

## Data Availability

Data are available from the corresponding author upon reasonable request.
